# Adjusting an Available Online Peer Support Platform in a Program to Supplement the Treatment of Perinatal Depression and Anxiety

**DOI:** 10.2196/mental.5335

**Published:** 2016-03-21

**Authors:** Amit Baumel, Stephen M Schueller

**Affiliations:** ^1^ The Feinstein Institute for Medical Research Manhasset, NY United States; ^2^ Department of Preventive Medicine Northwestern University, Feinberg School of Medicine Center for Behavioral Intervention Technologies Chicago, IL United States

**Keywords:** online, peer, support, perinatal, postpartum, depression, anxiety

## Abstract

**Background:**

Perinatal depression and anxiety are common and debilitating conditions. Novel, cost effective services could improve the uptake and the impact of mental health resources among women who suffer from these conditions. E-mental health products are one example of such services. Many publically available e-mental health products exist, but these products lack validation and are not designed to be integrated into existing health care settings.

**Objective:**

The objective of the study was to present a program to use 7 Cups of Tea (7Cups), an available technological platform that provides online peer (ie, listener) based emotional support, to supplement treatment for women experiencing perinatal depression or anxiety and to summarize patient’s feedback on the resultant program.

**Methods:**

This study consisted of two stages. First, five clinicians specializing in the treatment of perinatal mood disorders received an overview of 7Cups. They provided feedback on the 7Cups platform and ways it could complement the existing treatment efforts to inform further adjustments. In the second stage, nine women with perinatal depression or anxiety used the platform for a single session and provided feedback.

**Results:**

In response to clinicians’ feedback, guidelines for referring patients to use 7Cups as a supplement for treatment were created, and a training program for listeners was developed. Patients found the platform usable and useful and their attitudes toward the trained listeners were positive. Overall, patients noted a need for support outside the scheduled therapy time and believed that freely available online emotional support could help meet this need. Most patients were interested in receiving support from first time mothers and those who suffered in the past from perinatal mood disorders.

**Conclusions:**

The study results highlight the use of 7Cups as a tool to introduce accessible and available support into existing treatment for women who suffer from perinatal mood disorders. Further research should focus on the benefits accrued from such a service. However, this article highlights how a publicly available eHealth product can be leveraged to create new services in a health care setting.

## Introduction

### Perinatal Depression and Anxiety

Depression and anxiety during pregnancy or within the first 12 months after delivery (perinatal depression and anxiety) are common and debilitating conditions. More than 15% of women experience symptoms of depression and anxiety during this period [1-3]. Depression and anxiety have comparable impacts on quality of life [4] and are associated with high levels of lost work productivity and increased medical costs [5,6]. Untreated perinatal depression and anxiety carry several additional risks, including obstetric complications, puerperal pathologies, and increased psychopathology in childhood or adolescents [7-9]. Overall, psychosocial treatments appear to be preferred to pharmacotherapy [10-12]. These treatments do not confer any exposure risks to the babies, for example, [13,14], and the availability of emotional and social support during perinatal period has a significant role in the prevention and recovery from perinatal mood disorders. Research has shown, for example, that low-income pregnant women with higher quality of social support tend to experience less postpartum depression [15], and that even brief interventions of postnatal support groups are effective in reducing the symptoms of postpartum depression [16]. Several barriers, however, to receiving such services present during the perinatal period including lack of time, stigma, and childcare [17]. Overcoming these barriers requires novel resources that expand potential models of delivery [18], which can offer a variety of services to meet each person’s individual needs and preferences.

One new model of delivery is technology-based mental health services. Technology-based mental health services offer a host of features that can address several of these barriers and might be particularly useful among new and expectant mothers. First, technology-based interventions move the intervention outside of traditional clinics and into people’s households. Services can be available 24/7, on demand, which might be useful for women whose demanding schedules often offer only brief pockets of availability throughout the day. They can be less stigmatizing and the use of peer support embedded with these interventions could help normalize the experience of perinatal depression and anxiety. Indeed, many women express interest in the use of Internet-based health care resources during the perinatal period [19]. Furthermore, Internet-based resources for perinatal depression and anxiety have demonstrated feasibility and efficacy [20-25]. For example, a pilot trial of an interactive guided Web-based intervention for mothers with postpartum depression found significant reductions in depressive symptoms with 77% reporting clinically important improvement on the Patient Health Questionnaire [20]. In a randomized controlled trial comparing computerized behavioral activation for postpartum depression to treatment as usual (limited access to the Internet website), medium to large effect sizes favored women who received the computerized treatment on scores of depression (*d*=0.87) and anxiety (*d*=0.59) [21]. In addition, a randomized control trial investigating therapist-assisted online therapy for postpartum depression found greater reductions in depressive symptoms from participants receiving the intervention compared to a waiting list condition [22]. Finally, a study investigating the feasibility of an Internet-facilitated intervention for disadvantaged mothers with elevated symptoms of depression found greater reduction in depressive symptoms for participants in the intervention group compared to waiting list condition [23].

### Leveraging eHealth Products to Enhance Care

The promise of technology-based interventions in enhancing care has led to an expansion in the number of available e-mental health products in recent years. For example, a 2015 report identified over 165,000 health applications (apps) currently available on the leading consumer app marketplaces (Apple iTunes and Google Play) [24]. This is over twice as many as were available two years prior, with mental health apps making up the largest portion of these apps [24]. Despite the large number of apps available, very few have empirical support [25,26], and many health apps lack basis in empirically supported principles [27,28].

In light of this, a significant gap exists between the interest and availability of e-mental health products and the current evidence supporting their benefit. As such, many researchers are developing new e-mental health products to be subjected to randomized controlled trials, however, very few of these products then become available for consumers or implemented within health care settings. For example, a recent review of mental health apps for depression identified 4 trials assessing 3 apps for depression [25], but none of these apps are available for consumer download.

It is worth noting, however, that just because an e-mental health product has not been subjected to a randomized controlled trial, does not mean it does not have some sound behavior change principles or that it is not beneficial. Given the consistently changing nature of software and the technological environment, it has been suggested that behavior change principles are the more important unit of analysis than the apps themselves [29]. As such, moving e-mental health products into practice requires research and information to guide decision making in this regard. Boudreaux et al [30] suggest several practical steps for health care professionals to evaluate and select publically available apps including piloting apps and eliciting feedback from target end users. Similarly, Chan et al [31] highlight three dimensions including usefulness, usability, and integration and infrastructure. Although these steps and guidelines do not establish the efficacy of the e-product, they provide useful information about how such products might be beneficial for end users and integrated into existing care settings.

Furthermore, given the number of available eHealth care products, rather than developing and evaluating completely new products, a more efficient use of resources might be to develop additional scaffolding to make products more in line with evidence-based practices and needs of particular health care settings. This approach could also ensure that the resultant product or program can stay relevant to the current technological environment, which is a major challenge in this field [32]. Research along these lines could also uncover information about principles present in these programs or products, which could still guide subsequent research and development.

### The Current Study

This study presents a program to use 7 Cups of Tea (7Cups), an available technological platform that provides online peer-based emotional support, to supplement treatment for women experiencing perinatal depression or anxiety. 7Cups was chosen for this study because its solution enables to scalably train and engage interested individuals with those who seek their support [33], and also due to the high volume of available volunteers on the platform [34], which open new avenues for receiving peer-based emotional support in real world settings [35]. In this paper, we present the development process required to adapt the online emotional support platform for use as an adjunct to treatment in an existing health care setting and gather patient’s feedback on this program. We follow existing guidelines on key processes and factors to evaluate when considering existing eHealth products [30,31].

## Methods

### Adapting and Evaluating 7 Cups of Tea

The process of adapting and evaluating 7Cups was completed in two iterative stages. The first stage enlisted clinicians to identify program modifications necessary to use 7Cups to supplement existing treatment resources. This stage included providing these clinicians an overview of 7Cups, and discussing required safety practices and program modifications. In the second stage, patients with perinatal depression or anxiety used the platform for a single session and provided their evaluation of usefulness, usability, and impressions of the program.

### Stage I: Adjusting 7 Cups of Tea to Supplement the Treatment for Perinatal Mood Disorders

#### 7 Cups of Tea Overview

7Cups provides free, 24/7, emotional support to users through a Web- or app-based messaging system [33]. Volunteers, referred to as “listeners”, provide emotional support by receiving and answering chat requests from users. Before being able to respond to chat requests, listeners are required to complete a computerized training course on active listening, which includes video, text, and quiz components.

**Figure 1:**
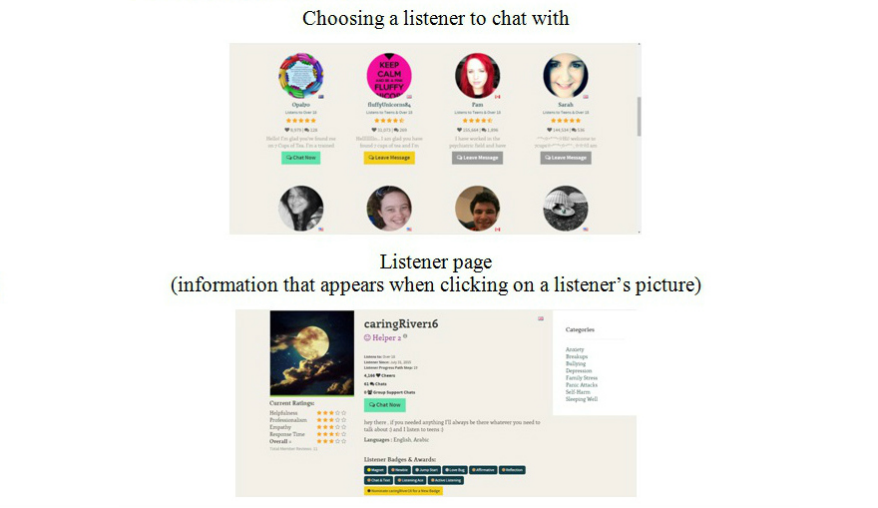
Listeners' list and information page.

Upon logging into the platform, users can choose who they would like to chat with from a list of available listeners (Figure 1 shows this). Users and listeners are identified using anonymous user names. The platform provides some information about listeners including their country, age, group they will listen to, preferred topics for chats (eg, parenting), and experience on the platform (eg, number of chats conducted). Listener’s average users’ ratings in several domains (eg, helpfulness, professionalism) are displayed on a 5-point scale. A previous study on 7Cups found high levels of user satisfaction with the support provided by listeners [36].

#### Safety Practices

**Figure 2:**
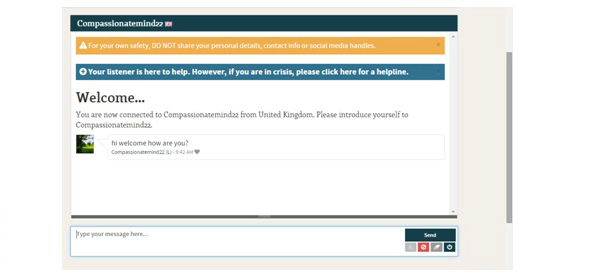
Beginning the chat on the 7 Cups of Tea (7Cups) platform.

Safety practices draw from recommendations for Internet-based intervention research and cover areas of system security, online safety, and clinical safety [37]. 7Cups online and system security was reviewed and approved by the IT (ie, information technology) risk management team of Feinstein Institute for Medical Research, part of NS-LIJ Health System. In terms of clinical safety, main concerns are confidentiality and ensuring users are not using 7Cups as a crisis management tool. In service of confidentiality, both 7Cups users and listeners are anonymous. In order to ensure that 7Cups is being used appropriately, users must confirm that they are not in a crisis situation before beginning to chat. Banners on the chat window ask users to refrain from providing personal or identifying information, and provide information regarding help for crisis situations (Figure 2 shows this). Listeners are also directed to refer users to more intense programs or other resources in cases of need.

#### Program Adjustments Based on Clinicians’ Feedback

Feedback was elicited during a group discussion involving five clinicians specializing in the treatment of perinatal depression (2 psychiatrists, 3 psychotherapists) facilitated by the first author (AB). The aim of the discussion was to gather feedback regarding the ways 7Cups could complement treatment, and to understand clinicians concerns regarding the use of 7Cups as a supplement to existing treatment. Clinicians were instructed that this discussion was part of a larger program to refer patients to use 7Cups while undergoing treatment for perinatal mood disorders, and that their feedback could help influence this program.

At the beginning of the discussion, the group facilitator (AB) demonstrated the features of 7Cups, explained the training 7Cups listeners receive, and how people use the 7Cups program. During this demonstration clinicians could ask clarifying questions. Clinicians were then asked to discuss how 7Cups and this kind of support could complement the treatment they currently deliver, provide recommendations for additional training that would help listeners support people who suffer from perinatal mood disorders, and report any concerns regarding the use of online, peer-based, emotional support. During the discussion, the group facilitator took notes, asked for clarifications, encouraged members to expand on points raised by others, and finally read the points gathered by the group to examine participants’ agreement. After presenting the main points, the facilitator asked participants if any additional comments or points needed to be considered.

#### Clinicians’ Suggestions

In the group discussion, clinicians stated that online emotional support could be beneficial to individuals who suffer from perinatal mood disorders by making support accessible immediately during times of need. Additionally, although many patients may have social support, that support network might be already taxed by helping women cope with stressors associated with pregnancy and the birth of a child. As a result, clinicians noted that in many cases, these patients do not have opportunities to share their feelings during the week and receive emotional support. As such, introducing 7Cups as an additional outlet for support could be beneficial. The group also noted that 7Cups provides this support online without requiring women to travel to the clinic to receive peer support. Time and travel are two barriers noted by the group and they saw value in overcoming these barriers.

The group suggested that listeners could be most helpful by: (1) providing active listening, (2) providing nonjudgmental support, and (3) pointing users to additional resources when relevant. The group also suggested that patients might desire to receive support from listeners who have personal experience with perinatal mood disorders.

The group emphasized the importance of educating patients who would use the platform to set appropriate expectations. This includes: (1) the difference between psychotherapy and emotional support, and how to appropriately utilize emotional support and (2) the inability of listeners to deal with crisis situations. Finally, the group also emphasized that listeners should be provided with basic information on perinatal depression and anxiety, as they are not trained experts in this area.

Based on clinicians’ feedback, guidelines for referring patients to use 7Cups as a supplement for treatment of perinatal depression and anxiety were created, and a training program for listeners was developed.

#### Guidelines for Referring Patients to Use 7 Cups of Tea to Supplement Treatment

Clinicians’ comments suggested guidelines to address potential safety concerns and to ensure that users have the proper education about how and when to use the platform. For the purposes of patient recruitment and referral, and in accordance with Internet-based intervention studies that relate to safety concerns [38,39] the following guidelines were created:

1. Patients with suicidal or homicidal intent will not be referred to use the online emotional support.
2. Patients with psychotic or manic symptoms will not be referred to use the online emotional support.
3. Patients who require hospitalization will not be referred to the online emotional support.
4. Patients using the platform will have to confirm while beginning to chat that they are currently not suicidal or homicidal (already embedded).
5. Patients will receive clear information (embedded in the platform and in program tutorial) that listeners cannot provide any support for emergency purpose. Helpline information will be provided on the platform for use in crisis situations.
6. Patients will receive clear information about the difference between the emotional support and treatment as psychotherapy, and about appropriate use cases.

#### Training of Listeners

**Figure 3:**
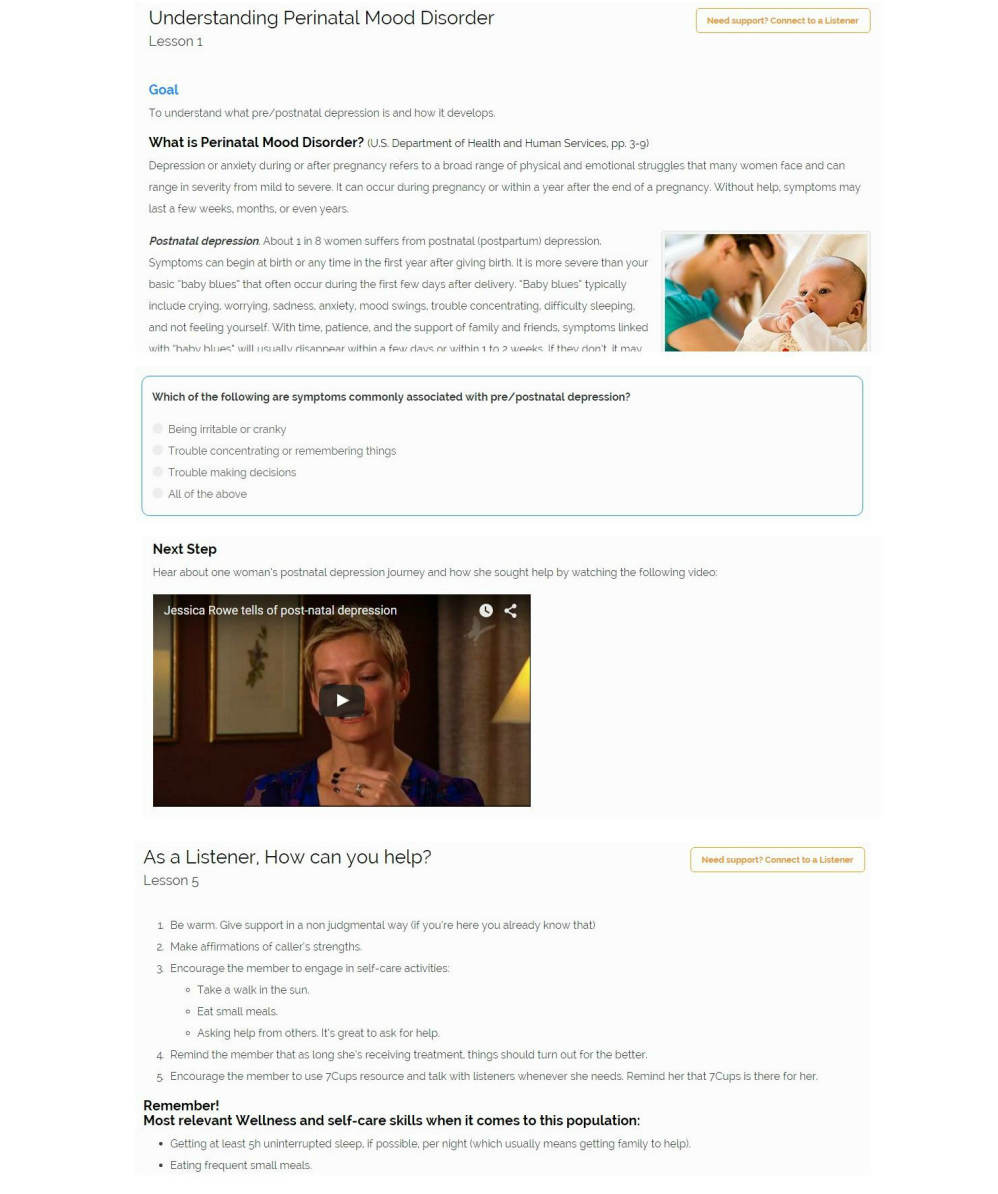
Sample screenshots of listener's computerized training.

A computerized training course was developed to provide listeners with relevant information for supporting women who cope with perinatal mood disorder. The specialized training made use of features present in the 7Cups training modules consisting of text, video, and quiz components. A psychologist (AB), a psychiatrist (AT), and 7Cups staff including the community manager, collaborated to create this training (Figure 3 shows sample screenshots).

This training included five lessons: (1) “understanding perinatal mood disorder”, explaining the illness, symptoms, prevalence, and course; (2,3) “how to feel better”, providing basic wellness and self-care skills and accessible resources for those suffering from perinatal mood disorders (eg, postpartum support international); (4) common misperceptions regarding perinatal mood disorder (eg, perinatal depression happens always right after childbirth); and (5) guidelines for the supporter (eg, “Encourage the member to engage in self-care activities: Take a walk in the sun. Eat small meals”.).

An invitation to complete this training course was sent to 7Cups listeners who had received the “verified listener” badge. A listener obtains this badge by completing a test chat with another listener and providing a positive and supportive experience. There were 64 listeners that signed up for the training in a one-week period (March 31 to April 6, 2015) and 46 completed it. These listeners were available to provide emotional support for women participating in stage II of this study.

### Stage II: Patient Feedback

#### Participants

In the second stage, nine patients with perinatal depression or anxiety disorders were recruited from the Adult Outpatient Department in The Zucker Hillside Hospital. Outpatient clinicians were provided inclusion and exclusion criteria and indicated which of their patients would be eligible. Eligible patients required a diagnosis of perinatal depression or anxiety as indicated by their psychiatrist and recorded in their electronic medical record. Participants’ ages ranged between 25 and 34 (M =28.9, SD=2.6), and most of them suffered from postnatal mood disorder (see Table 1 for demographics and diagnostic information).

**Table 1:** Demographics and clinical diagnoses.

Participant	Age	Racial background	Clinical diagnosis			
			Antenatal	Postnatal	Depression	Anxiety
1	29	White		X		X
2	29	White		X		X
3	29	Multiracial	X		X	
4	25	Black/African American		X	X	
5	29	Unknown		X	X	
6	31	White		X	X	X
7	27	White		X		X
8	34	Unknown		X		X
9	27	Unknown	X			X

#### Procedure

**Figure 4:**
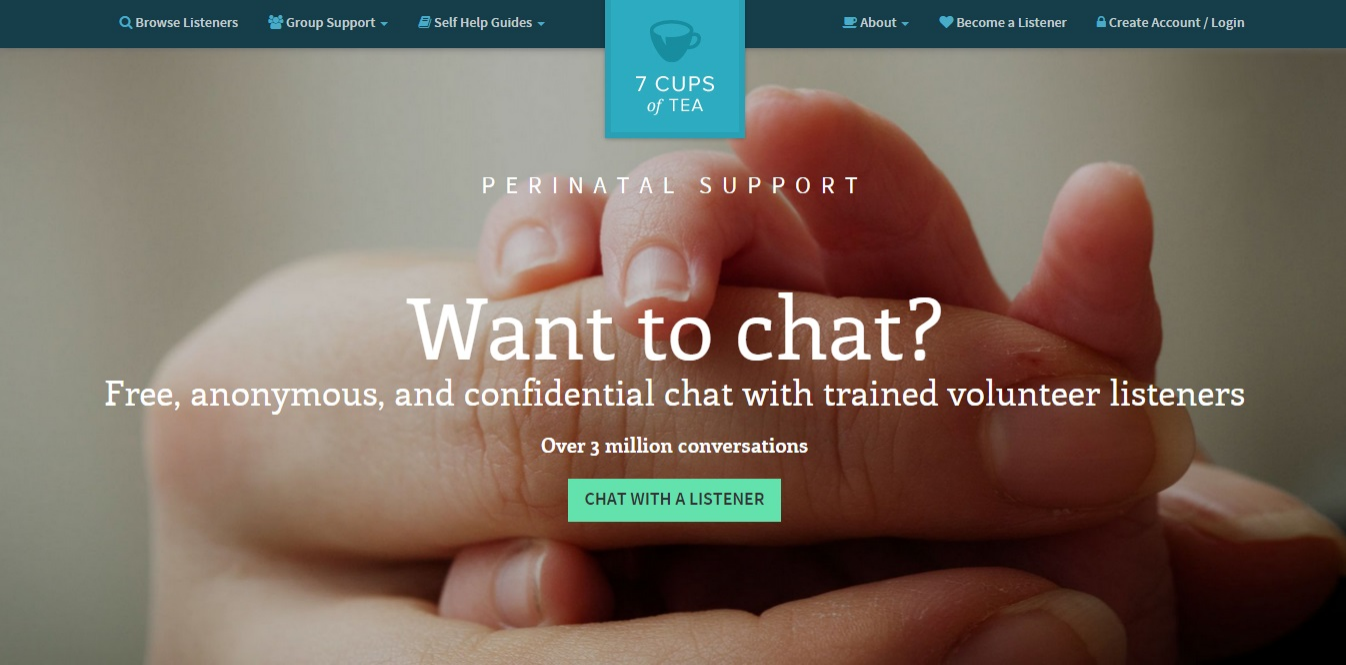
Perinatal mood disorders landing page.

Participants who met criteria were offered the opportunity to take part in this study by their clinician. Interested individuals provided their contact information and received a phone call from the first author (AB). On that call, the first author described the study’s purpose and general procedure and obtained informed consent for study participation. Participants who consented to participate received an email with details regarding the study procedure. Participants were asked to examine 7Cups by conducting at least one chat on a day of their choosing between the hours of 7 and 10 p.m. A time window was required in order to ensure that listeners who completed the perinatal mood disorder training module would be available to chat. Clinical staff recommended 7-10 p.m. as a time that might be ideal for listeners as well as users taking care of a new baby. Participants received an email with instructions how to use the platform including information, screenshots, and a detailed description of how to chat with listeners and were asked to contact the first author (AB) if they needed additional guidance. Participants accessed 7Cups through a specialized Web page (Figure 4 shows this), which limited the available listeners to those who completed the training program. Participants were asked to inform the first author when they had completed a chat session. At that time, they received a survey link via email asking them to report on their experience. Participants received a US $25 Amazon gift card compensation for their participation. The Feinstein’s Institutional Review Board approved study procedures.

#### Measures

The survey consisted of Likert-type questions and free response open-ended questions. Five point Likert-scale measures addressed attitudes both toward the 7Cups platform and the listeners providing support. There were 8 items that assessed usability, usefulness, and intention to use and recommend 7Cups and were adapted from established measures including the USE [40], Behavioral Intention to Use [35], and Word of Mouth Communication [41,42]. Attitudes toward the listeners were measured using four items that were used in a previous study on 7Cups [36], adapted from measures of therapeutic alliance [43-45]. Minor modifications to existing questions were made to ensure they were relevant to 7Cups users (eg, “therapist” was replaced by “listeners”) and we consulted specialist health providers to confirm that modified questions were clear and understandable. Open-ended questions queried participants about additional aspects including general remarks regarding the program, in what ways (if any) using this support may complement treatment, preferences around use (ideal listener, times of use), and suggestions for improvements.

Data analysis included descriptive statistics of the multiple-choice questions, and an inductive thematic analysis of the answers to open-ended questions regarding general experience and complementing treatment. The latter was based on the six-phase method suggested by Braun and Clarke [46] which includes: familiarization with data, generation of initial codes, searching for themes among codes, reviewing themes, defining and naming themes, and producing the final report.

## Results

### Satisfaction and Acceptance of the Online Emotional Support

Responses to the rating scales are presented in Table 2. All participants rated the emotional support program as usable and useful. Attitudes toward the listeners were extremely positive with 97% (35/36) of these responses being either agree or strongly agree. The highest ratings came on items related to the perceived helpfulness of the program including: “This kind of support can be helpful for me” (8/9 = strongly agree) and “I can see how in a certain amount of time after chatting with listeners, people can feel better” (8/9 = strongly agree). Finally, although all participants indicated that they would use and recommend 7Cups in relevant cases, only a minority indicated they would like to join 7Cups as listeners when they feel better.

**Table 2:** Participant usability, usefulness, intention to use and recommend, and attitudes toward the listeners’ ratings (n=9).

		Strongly disagree	Disagree, n (%)	Neutral, n (%)	Agree, n (%)	Strongly agree, n (%)
**Usability**						
	I found the program accessible and easy to use.	0	0/9 (0)	0/9 (0)	5/9 (55)	4/9 (44)
**Usefulness**						
	I liked to use this service.	0	0/9 (0)	0/9 (0)	4/9 (44)	5/9 (55)
	This kind of support can be helpful for me.	0	0/9 (0)	0/9 (0)	1/9 (11)	8/9 (88)
**Intention to use and recommend**						
	I would recommend this program to people who suffer from perinatal mood disorder.	0	0/9 (0)	0/9 (0)	2/9 (22)	7/9 (77)
	I would recommend friends to use 7Cups.	0	0/9 (0)	0/9 (0)	7/9 (77)	2/9 (22)
	I would recommend on using 7Cups to people who suffer from mental health difficulties.	0	0/9 (0)	0/9 (0)	5/9 (55)	4/9 (44)
	I would probably use 7Cups in the future if needed.	0	0/9 (0)	0/9 (0)	5/9 (55)	4/9 (44)
	When I will feel better, I would like to join 7Cups as a listener.	0	4/9(44)	3/9 (33)	1/9 (11)	1/9 (11)
**Attitudes toward the listeners**						
	I felt the listeners cared about me as a person.	0	0/9 (0)	1/9 (11)	4/9 (44)	4/9 (44)
	Listeners can do a good job in supporting people who suffer from perinatal depression/anxiety.	0	0/9 (0)	0/9 (0)	4/9 (44)	5/9 (55)
	I believe these listeners can help people who suffer from mental health difficulties.	0	0/9 (0)	0/9 (0)	7/9 (77)	2/9 (22)
	I can see how in a certain amount of time after chatting with listeners, people can feel better.	0	0/9 (0)	0/9 (0)	1/9 (11)	8/9 (88)

### Thematic Analysis of Open-Ended Responses

Overall, most participants expressed a positive experience related to using the platform and indicated that easily available emotional support could complement treatment by enabling them to receive just in time outlets for stress and emotions (see Table 3 for an overview of themes found in the analysis). Approximately half of participants suggested that this service could complement treatment by providing support when the clinicians are not available, and the same proportion of participants indicated that this program might benefit them by providing an additional support person. Additionally, approximately half of participants noted a benefit of this program was it enables easy access to emotional support. A participant did comment negatively on feeling that it is difficult to benefit when communicating on an online platform: (“It was fine, but I didn't love it. Personally, I enjoy the bond forged by an in-person meeting. I liked the service in general, but do not feel that the chat that I had was beneficial to my overall anxiety”.).

**Table 3:** Themes found in participants’ general remarks regarding the program and answers to the ways using this support may complement treatment.

Theme	n	Examples
Positive experience. A positive experience related to using the program.	8	“It was nice to discuss the experience I went through”. “Very helpful for me”.
Immediate outlet in a moment of need. Enabling to receive just in time outlet for stress and emotions.	8	“I think if one is in the throes of anxiety or a severe depression, it can be very helpful to talk things out in the moment”. “Immediate feedback and immediate response when you are dealing with high levels of depression or anxiety”.
Being there when the therapist is not available.	5	“Doctors and therapists are not always around, especially late at night. This is a great service for mom's to be able to use during off hours”.
Providing additional support	4	“Another person validating my feelings are ok”. “It would reinforce the support”.
Accessible. Easy to access and use.	4	“It's easy when you have a young child that you don't need to leave the home or be on the phone”.
Nonjudgmental approach.	3	“I think it's good for people to have someone to talk to that is not seeing them so they don't feel judged at all. Therapists or loved ones are good, but there is still a certain level of feeling judged”.
Providing hope and comfort.	3	“It's a nice feeling knowing there's someone out there who will listen”.

### Suggested Modifications

Participants desired increased availability from the listeners, noting that it would be helpful to have more listeners to choose from at any given time. A participant wanted to be able to designate a preferred listener so they would not have to repeatedly explain their story. Another participant requested email notifications when responses within the platform were replied to in order to eliminate the need to log in and check within the platform. Another participant requested for listeners’ ages to be displayed, as she chatted with a listener for some time before learning the listener was fourteen years old.

### Preferred Listeners’ Identity

Participants were specifically asked preference for gender of the listener. There were six participants that indicated a preference for female listeners, whereas the other 3 had no preference between men or women. In the open-ended responses, 5 participants wrote that they prefer the listener will have personal experience with depression or anxiety (“Someone who has experienced the same kind of anxiety or depression that I have, so they can understand and be supportive”.). A participant expressed preference for someone who has experience dealing with anxious people or first time mothers.

### Preferred Times to Chat

Participants were asked to indicate all times in which they would prefer using 7Cups. There were six participants that identified the evening (6-10 p.m.) or nights (10 p.m. or later) as preferred times to chat with listeners. There were two participants who identified both days and nights as preferred times. A participant identified the day as the only preferred time to use this service. A participant wrote, “I honestly wish this service was available 24/7. Being a parent is a 24/7 job”.

## Discussion

### Principal Findings

Following clinicians’ feedback and program adjustments, patients with perinatal depression and anxiety reviewed 7Cups and chatted with listeners. They found the platform easy to use, useful, and indicated they could see themselves using 7Cups and recommending it to other women who suffer from perinatal depression or anxiety. Patients also viewed the listeners quite positively.

These findings suggest that women who suffer from perinatal mood disorders recognize a need for help outside the scheduled therapy time and perceive online emotional support as a potential tool to meet this need. Although patients’ preferred time windows for chats lined up with the providers’ recommendations, it seems that participants wanted much more availability and perceived the listeners’ availability as one of the main program advantages. This finding converges with the Pugh et al study [47] showing that one of the main perceived advantages of a therapist assisted online program for women who suffer from postpartum depression revolves around the program flexibility and accessibility, due to the mothers’ need to manage themselves around the child care schedule.

It also seems that patients who suffer from perinatal depression or anxiety are interested in receiving support from people either “like them” (women, people with experience with depression, anxiety, first time mothers) or those who have experience with people like them. Accordingly, such platforms should find appropriate ways to inform users regarding which volunteers have previously experienced these disorders, and attempt to recruit more women who had personal experience with perinatal depression or anxiety. Potential concerns include confidentiality and stigma on the part of the volunteer; however, overcoming these concerns could be a solvable problem in the design and implementation of future platforms.

It is worth noting that the majority of the study’s participants did not see themselves volunteering in this kind of platform in the future. It could be useful, thus, to explore why women, who found this platform helpful, would be hesitant to volunteer for such a platform. It is plausible that these women feel overwhelmed by the demands of caring for a new baby and soliciting mothers with older children, who had experienced perinatal depression or anxiety disorder, would be a better target. Another possibility is that women might be more likely to volunteer after they had received extended support from this program and experienced a reduction in the mood and anxiety symptoms such that they would feel better able to support someone else through the process. It might also be worth exploring methods that allow people to receive and provide support at the same time, making the platform more scalable as additional users join, for example, [48]. Another solution to overcome the limitation in the number of volunteers would be for a single trained peer to offer support to a group of users. For example, studies have demonstrated that online group support can be beneficial for women experiencing postpartum depression [49], and depressive symptoms following breast cancer diagnosis [50]. With these considerations taken into account, one should note that in less than one week, 46 listeners were trained and then became available for this study. In fact, it is likely we could have trained more listeners, as the training is an automated and nonconsumable [51] resource. However, we choose not to do so given the small number of study participants, as we believed that recruiting listeners without providing them the opportunity to chat could contribute to dissatisfaction and burn out on the part of the listeners. Nevertheless, we cannot comment on how many listeners would be required to provide opportunities to chat around the clock. We limited our supply such that it would not outstrip demand, but given the large burden of perinatal depression and anxiety (and other similar mental health conditions), it could be possible that demand would be such that additional considerations would have to be made to recruit more listeners.

Participants would also appreciate a better display of listeners’ characteristics and clear guidance on how to choose a listener based on these characteristics. A participant chatted with a very young listener, another participant was under the impression that she would have to repeatedly explain her story during each chat, and while some participants believed men could provide suitable support, others did not. Based on this feedback, it seems that different women might prefer different listeners. Choosing a listener is a critical step in using such a platform. Examining the listeners’ characteristics and qualifications prior to the chat can provide appropriate expectations on whether that listener would be a good fit. It seems that platforms such as 7Cups could benefit from adding tutorials to educate users how to identify the most suitable listener for their needs, and from presenting relevant information such as listeners’ age in a more prominent manner.

It is worth noting that not all patients perceived this platform as beneficial. Indeed, 7Cups served only as a tool to provide emotional support. It might be helpful to also integrate more didactic information and resources to teach skills to cope with mood and anxiety. The clinicians’ feedback called for increased knowledge of skills and resources for the listeners to recommend to users and this was included in the training program created. Digital tools such as mindfulness training exist [52,53] and could be impactful if provided to users.

Findings from this study should be considered in light of its limitations. First, feedback came from a few number of patients. However, given the formative stage of this research, significant problems and needs can be identified using only a few users. In the case of usability concerns, most problems can be uncovered using only five users [54]. Second, only a few listeners were trained to support the program with limited availability to provide support (only 7-10 p.m.). This made sense as this trial deployment recruited a few people to use the program and listeners available throughout the day would be used rarely and might become bored or unmotivated. A larger deployment with more users would necessitate more listeners, however, this could also expand availability throughout the day. Third, the program to support women with perinatal depression or anxiety was designed to supplement treatment, and thus, the results do not speak to whether this support is sufficient for women who suffer from these conditions, but do not receive formal mental health care. Finally, listeners in this project went through additional training and were required to have received the verified listener badge. Thus, not only had these listeners already indicated being a subset of listeners (through receiving the badge), but their willingness to complete the training may also differentiate them. The positive ratings in our study, including the “attitudes toward the listener”, may not apply to chats completed with other listeners. Indeed, quality of peer supporters within this and other programs is a likely determinant of successful outcomes, which is one reason why the training program was included in our study.

### Future Directions

The aim of the current paper was to present the development of a program to supplement treatment for perinatal mood disorder by leveraging an existing online platform and eliciting patients’ opinions of this program. This study was motivated by a desire to implement such a program into a health care setting, introducing a novel form of support without placing added burden on health care providers. More research needs to address implementation into practice and evaluate efficacy and feasibility in real-world settings [55]. Leveraging the online emotional support provided by 7Cups listeners to complement ongoing treatment for perinatal depression or anxiety is a powerful example of such efforts.

Efforts are currently underway to expand the use of 7Cups for additional patients and to examine the utilization, satisfaction, and clinical outcomes of patients using this service. Based on the results of this study, modifications include training more listeners, recruiting listeners with personal experience of perinatal mood disorders, providing group support tools, integrating relevant evidenced-based self help tools within the platform, and appropriately presenting relevant information about the listener.

Furthermore, similar investigations could evaluate the feasibility and impact on providing such a resource to women with perinatal depression and anxiety who are not receiving formal mental health care resources. Many women who suffer from these conditions are not able to receive formal resources for a variety of reasons (access, cost, time) and the resource examined in this study or similar tools could provide some benefit in these cases.

### Conclusions

The results of this study highlight the promise of 7Cups as a tool to introduce accessible, freely available peer support into existing treatment settings. Along with other studies that have demonstrated that peer support is an effective tool to prevent perinatal depression via phone [56] and is generally acceptable by women who suffer from postpartum depression [57,58], 7Cups could meet several of the needs of women seeking treatment for perinatal depression or anxiety. These findings are also congruent with the Griffiths et al study [59] showing that peer support delivered online is perceived to provide a useful support for those who suffer from depression. This study also demonstrates how adjustments made to a publically available off-the-shelf product could increase its usefulness for a given health care setting. Given the high development costs of technological resources and evolving technological landscape, studies that use existing tools rather than developing and evaluating completely new products might be more likely to influence current clinical practices.

## Abbreviations

apps: applications
7Cups: 7 Cups of Tea
